# Precision Oncology in Older Cancer Patients: A Single-Center Experience

**DOI:** 10.3390/ijms252011322

**Published:** 2024-10-21

**Authors:** Meret Petitat-Berli, Marie Knufinke, Michèle Voegeli, Martina Sonderegger, Bettina Seifert, Elena Diana Chiru, Pirmin Haeuptle, Lisanne van’t Walderveen, Robert Rosenberg, Emanuel Burri, Svetozar Subotic, Fabienne Dominique Schwab, Vérène Dougoud-Chauvin, Heinz Unger, Kirsten Mertz, Loay Tahan, Marcus Vetter

**Affiliations:** 1 Center of Oncology and Hematology, Medical University Clinic Baselland, 4410 Liestal, Switzerland; 2 Center of Geriatric Medicine and Rehabilitation, Cantonal Hospital Baselland, 4101 Bruderholz, Switzerland; 3 Laboratory of Nutrition and Metabolic Epigenetics, Department of Health Sciences and Technology, ETH Zurich, 8092 Zurich, Switzerland; 4 Colorectal Cancer Center, Cantonal Hospital Baselland, 4410 Liestal, Switzerland; 5 Department of Surgery & Visceral Surgery, Cantonal Hospital Baselland, 4410 Liestal, Switzerland; 6 Gastroenterology and Hepatology, Medical University Clinic Baselland, 4410 Liestal, Switzerland; 7 Clinic of Urology, Cantonal Hospital Baselland, 4410 Liestal, Switzerland; 8 Gynecologic Cancer Center, University Hospital of Basel, University of Basel, 4031 Basel, Switzerland; 9 Clinic of Oncology, HFR Fribourg Hospital, 1708 Fribourg, Switzerland; 10 Institute of Pathology, Cantonal Hospital Baselland, 4410 Liestal, Switzerland

**Keywords:** next generation sequencing, precision oncology, older cancer patients, metastatic cancer

## Abstract

In the last two decades, next-generation sequencing (NGS) has facilitated enormous progress in cancer medicine, in both diagnosis and treatment. However, the usefulness of NGS in older cancer patients is unclear. To determine the role of NGS in older cancer patients, we retrospectively assessed demographic, clinicopathologic, and disease-specific data from 100 randomly selected cancer patients (any subtype/stage) who underwent NGS testing in 2020 at our institution and compared the treatment outcomes (progression-free survival [PFS] and overall survival [OS]) in the younger and older patient cohorts (A [n = 34] and B [n = 66]: age < 70 and ≥70 years, respectively). Overall, 27% had targetable mutations, and 8% received NGS-determined targeted therapy (45% and 19% of patients with a targetable mutation in cohorts A and B, respectively; *p* = 0.2), of whom 38% (3% of the whole cohort) benefited from the therapy (PFS > 6 months). The median OS (from diagnosis) was 192 and 197 weeks in cohorts A and B, respectively (*p* = 0.08). This pilot study revealed no significant age-stratified difference in the diagnostic approach and treatment strategy. A small, but relevant, proportion of the cohort (3%) benefited from NGS-determined treatment. Nevertheless, older cancer patients with targetable mutations less frequently received targetable therapies.

## 1. Introduction

In Switzerland, approximately 45,000 patients are diagnosed with cancer every year, and the 70–74 year age group is most frequently diagnosed with cancer [1]. Worldwide, approximately two-thirds of all cancer patients are older adults (age ≥ 65 years) [2]. In Switzerland, cancer is the second most common cause of death [3]. The treatment of cancer in Switzerland is organized into cancer centers, university hospitals, cantonal hospitals, regional hospitals, and private practices, and patients have broad access to cancer therapeutics, including off-label uses of uncommon and unapproved drugs. Article 71 enables physicians to prescribe off-label cancer drugs after receiving approval by the insurance company, and this treatment is reimbursable [4]. This aspect confers the advantage of broad access to cancer therapeutics that is based on the current oncologic literature and the latest FDA (Food and Drug Administration) and EMA (European Medicines Agency) approvals worldwide.

Recently, enormous progress has been made in the field of oncology in terms of both diagnostic and treatment options. A method that has contributed to this progress is next-generation sequencing (NGS)—a technology that enables rapid and accurate sequencing of many tumor genes. Although mainly used for research purposes until recently, in recent years, NGS has been increasingly used in routine cancer diagnostics owing to the decreasing cost and increased availability [5]. In oncology, NGS allows the identification of cancer-related genomic alterations in tumor cells, which improves the understanding of tumor biology and the tumor microenvironment and thereby plays an important role in cancer progression. This newer knowledge facilitates the development of novel therapeutics that target specific molecular targets in the tumor microenvironment and thus enable personalized, effective targeted treatment of patients with advanced cancer when standard therapies have been exhausted.

At the Cancer Center Baselland, Liestal, Switzerland, NGS is used for diagnostic and treatment purposes. Depending on the tumor entity (solid or non-solid), tumor tissue, and timepoint of tumor disease (at diagnosis or later), different NGS panels are used to determine different numbers of genomic alterations in the examined tissue. In most cases, only a small number of NGS-detected mutations are pathogenic and relevant for the currently available targeted therapies. However, variants of unknown significance may be relevant for therapy in the future [5]. Numerous studies with approved and off-label therapies are being conducted for individual mutations, and the findings are constantly evolving. Therefore, the discussion of NGS results at a molecular tumor board with experts from molecular biology, molecular pathology, and medical bioinformatics, as well as physicians with molecular cancer expertise, is of great advantage when the results and therapy recommendations are interpreted and selected according to the best evidence [6]. Furthermore, this approach is a method of precision oncology, which is a patient-centric approach to “find the right drug for the right patient at the right time” [7].

Until recently, the clinical benefit of NGS analyses was questioned [8,9,10,11], but real-world studies have increasingly demonstrated the significant impact of next-generation sequencing (NGS) in guiding treatment decisions for patients with metastatic cancer. Cohorts of patients who underwent NGS testing to identify actionable mutations often showed substantial benefits when treated with targeted therapies tailored to their genetic profiles. For instance, large national trials such as the TAPUR and NCI-MATCH studies provided strong evidence that NGS can uncover clinically actionable mutations, leading to the administration of targeted therapies that resulted in significant clinical responses, particularly in rare cancers with limited standard treatment options [12,13,14,15,16,17]. Additionally, a study from the Institut Curie in France showed that the integration of NGS-guided targeted therapies in patients with non-small cell lung cancer (NSCLC) significantly improved overall survival over the last two decades [18].

There is a limited knowledge of the use and effectiveness of NGS analyses and precision medicine in older patients with cancer. According to the NCCN Guidelines, older adults are defined based on functional status rather than chronologic age [19]. Age 65 years and older is generally considered the chronologic definition of an older adult, as this is the usual age of eligibility for Medicare benefits. In our institution, patients aged 70 years and older are admitted to geriatric medicine. We have therefore decided to speak of elderly patients from the age of 70 years onwards. Especially for this group of patients, who are most frequently affected by a new tumor or re-diagnosis, evidence-based treatment recommendations are rare because, owing to the inclusion and exclusion criteria, older patients are often underrepresented in clinical trials, including those of targeted agents [20]. Moreover, trials that specifically focus on the treatment of older patients are rare [21]. Therefore, the treatment of older cancer patients is particularly challenging due to comorbidities, cognitive impairment, frailty, loss of function, social isolation, etc. [22]. As these patients do not tolerate many standard tumor therapeutics because of their comorbidities, NGS findings could be very valuable for patients of this age group.

Despite the considerable data generated in the field of precision oncology in recent years, we suspect that these data remain underutilized, particularly in older patients with cancer. In addition, we suspect that older patients with cancer undergo fewer NGS examinations than younger patients and might be undertreated even after the identification of targetable mutations and the corresponding targeted agent.

Therefore, we conducted this study with an aim to examine the role of NGS in older and younger populations of patients with cancer at our institution.

## 2. Results

### 2.1. Results of Demographic, Clinicopathologic, and Disease-Specific Data

Patient characteristics are summarized in Table 1. In our cohort of 100 patients, 34 (34%) were <70 years old (cohort A) and 66 (66%) were ≥70 years old (cohort B) at the time of the NGS analysis in 2020. The median age at NGS was 72 years (range 35–89) overall, and 59 (35–69) years in cohort A and 76 (70–89) years in cohort B. The median age at primary diagnosis was 71 (29–89) years overall, and 56 (29–69) and 74 (48–89) years in cohorts A and B, respectively. Fifty-six percent of the overall patient population was female, with the same percentage in cohorts A and B.

Overall, our patients had a median number of 3 comorbidities (range: 0–10) overall, and 2 and 4 in cohorts A and B, respectively (both 0–10), and this difference was statistically significant (*p* = 0.02). Among the four main comorbidities, cardiovascular comorbidities were the most common (62% overall, 59% in cohort A, and 64% in cohort B), followed by pulmonary comorbidities (24% overall as well as in cohorts A and B), diabetes (21% overall, 18% in cohort A, and 23% in cohort B), and renal insufficiency (13% overall, 6% in cohort A, and 17% in cohort B).

Data on the ECOG Performance Status (ECOG PS) at diagnosis was missing in approximately one-third of the patients, and most of the patients with a documented ECOG PS had scores of 0 and 1 (77% of patients overall, 78% in cohort A, and 81% in cohort B); none of the patients had an ECOG PS score of 4. Information on the G8 status was unavailable for all patients.

**Figure 1:**
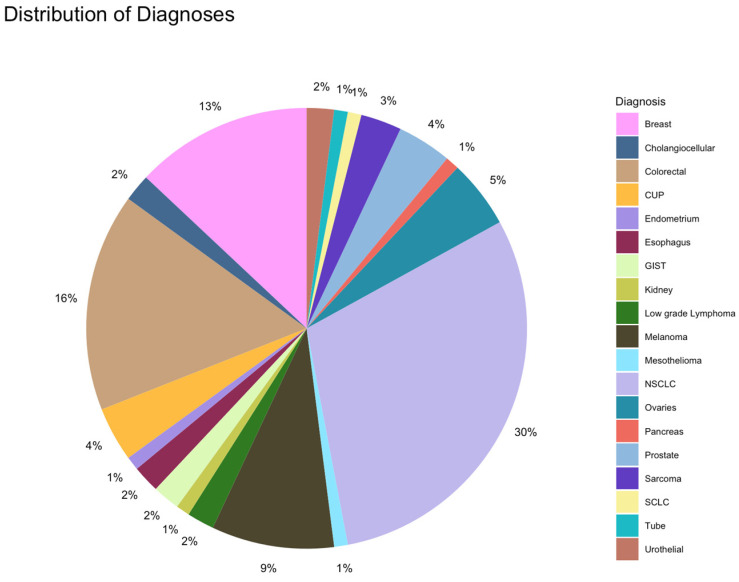
Distribution of diagnoses in the study cohort.

The distribution of diagnoses in the overall population is depicted in Figure 1. The most frequent tumor category in the overall population was NSCLC, followed by colorectal and breast cancers. In cohort A, colorectal cancer was more common than NSCLC (26% colorectal cancer, 18% NSCLC, and 18% breast cancer), whereas in cohort B, NSCLC was the most common tumor entity (36%), followed by colorectal cancer, breast cancer, and melanoma (11% each).

Overall, 55% of patients had a localized cancer stage at diagnosis, and this proportion was higher in cohort A (65%) compared with cohort B, wherein 50% of the patients had a localized cancer stage whereas the other 50% had metastatic cancer at diagnosis. The median (range) number of treatment lines was 2 (1–15) overall, 6 (1–12) in cohort A, and 2 in (1–15) cohort B, and the intergroup difference was statistically significant (*p* = 0.001).

Only 6 patients received >3 lines before NGS; these included 3 patients each in cohorts A (9%) and B (5%).

### 2.2. Results of NGS Analysis

The results of NGS analysis are summarized in Table 2.

**Figure 2:**
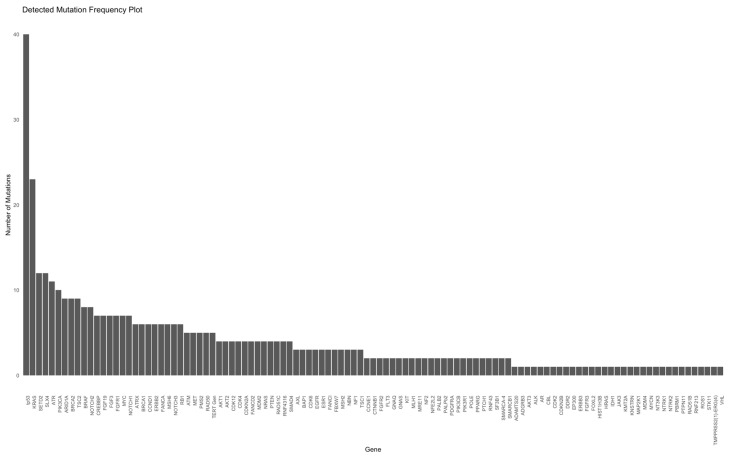
Frequency and name of all mutations.

**Figure 3:**
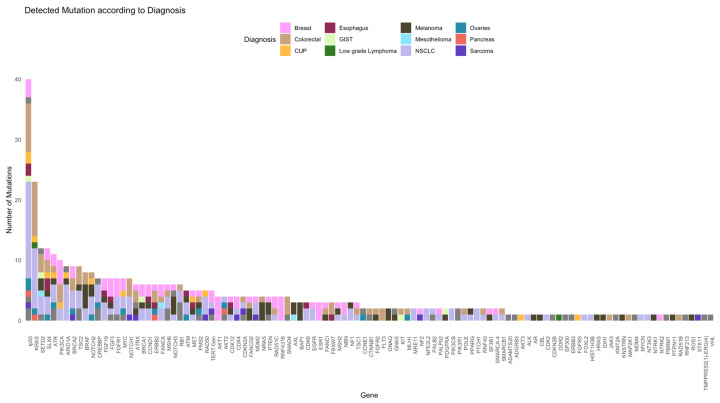
The mutations that were detected were stratified according to the diagnosis.

In this study cohort, NGS was performed in 55% and 45% at diagnosis and later stage, respectively. Patients in cohort A underwent NGS analyses more frequently at a later stage (59%) than those in cohort B (NGS at diagnosis, 62%). The Oncomine Comprehensive Assay Version 3 (OCAv3) was the most frequently used NSG panel (87% of the study cohort) and covered 161 of the most relevant cancer driver genes. A total of 109 different mutations were identified overall (71 and 94 in cohorts A and B, respectively). The five most commonly mutated genes were *tp53*, *KRAS*, *SETD2*, *SLX4*, and *ATR* (Figure 2 and Figure 3).

**Figure 4:**
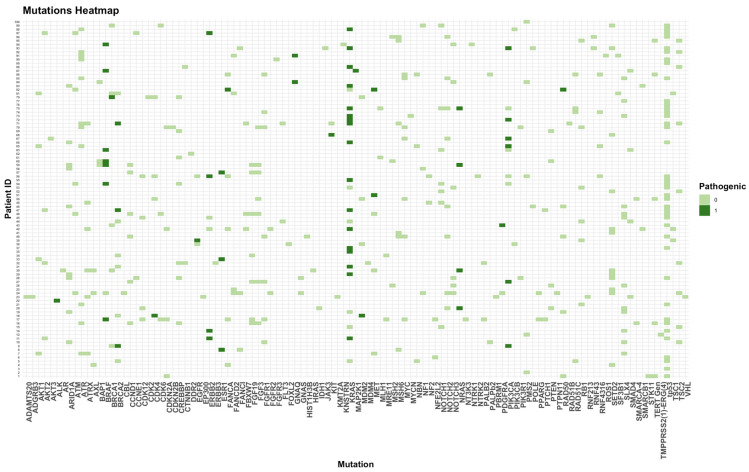
Heatmap of all pathogenic and not-pathogenic mutations. 0 = no pathogenic mutation; 1 = pathogenic mutation.

Overall, 38%, 9%, and 1% of the patients had 1, 2, and 3 pathogenic mutations, respectively (Figure 4). In cohort A, 12 (35%) and 4 (12%) patients had 1 and 2 pathogenic mutations, respectively. In cohort B, 26 (39%), 5 (8%), and 1 (1.5%) patient(s) had 1, 2, and 3 pathogenic mutations, respectively.

**Figure 5:**
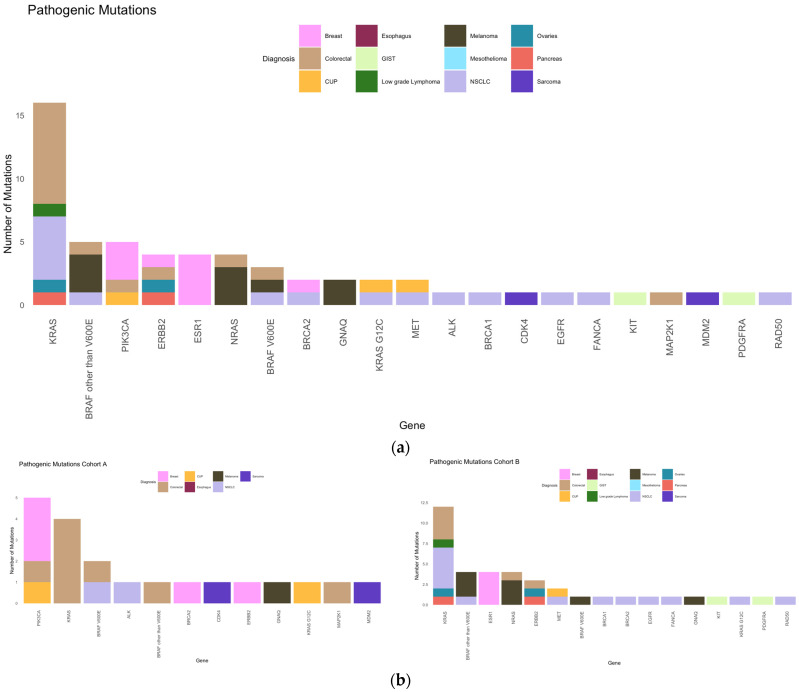
Pathogenic mutations. (**a**) Frequency of pathogenic mutations in our collective according to diagnosis. (**b**) Frequencies of pathogenic mutations in cohorts A (left) and B (right).

The most frequent pathogenic mutation (Figure 5) overall was *KRAS* (27%)*,* followed by *BRAF* other than *V600E* and *PIK3CA* (8% each), and *ERBB2, ESR1*, and *NRAS* (7% each). The most frequent pathogenic mutations in cohort A were *PIK3CA* (25%)*, KRAS* (20%), *BRAF V600E* (10%), whereas in cohort B, these included *KRAS* (31%)*, BRAF* other than *V600E*, *ESR1* and *NRAS* (each 10%), and *ERBB2* (8%).

**Figure 6:**
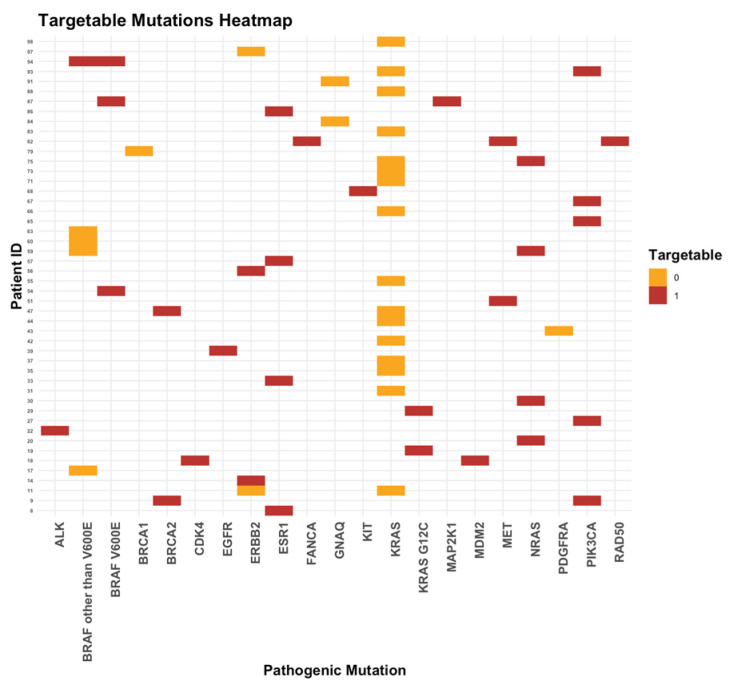
Targetable mutations.

Overall, 27% of our patients had targetable mutations (22%, 4%, and 1% of the patients had 1, 2, and 3 targetable mutations, respectively). In cohort A, 8 (23.5%) and 3 (9%) patients had 1 and 2 targetable mutations, respectively. In cohort B, 14 (21%), 1 (1.5%), and 1 (1.5%) patient(s) had 1, 2, and 3 targetable mutations, respectively (Figure 6).

**Figure 7:**
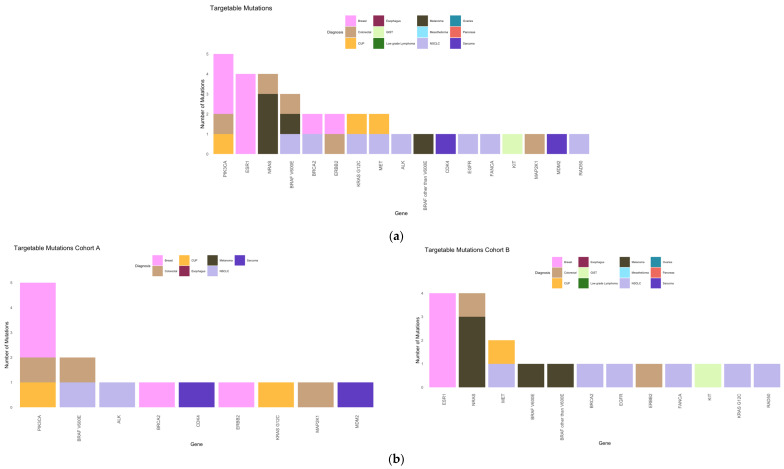
Targetable mutations stratified by the diagnosis. (**a**) Frequency of targetable mutations in the study cohort. (**b**) Frequency of targetable mutations in cohorts A (left) and B (right).

The most frequent targetable mutations were *PIK3CA* (15%), *ESR1* and *NRAS* (each 12%) overall; *PIK3CA* (36%) and *BRAF V600E* (14%) in cohort A; and *ESR1* and *NRAS* (each 21%) and *MET* (11%) in cohort B (Figure 7).

### 2.3. Discussion of Results at Molecular Tumor Board/Tumor Board

The details of most of the patients were discussed with the institutional tumor board. In 2020, only a minority of patients were discussed with the molecular tumor board of the university hospital in Basel.

### 2.4. Treatment Recommendations and Clinical Outcome

**Figure 8:**
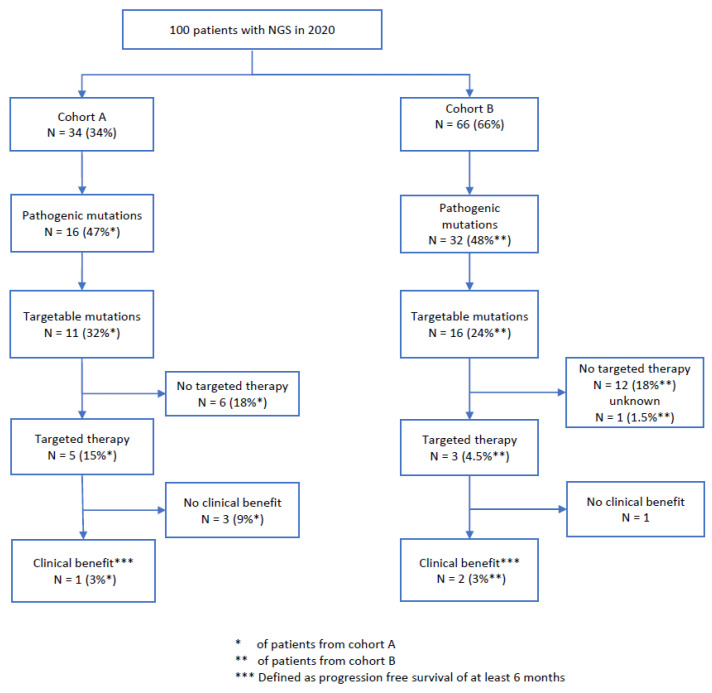
Flowchart for the clinical benefit of targeted therapy.

In total, 8 of our 27 patients (30%) with targetable mutations and 8% of all patients received a targeted therapy selected according to the NGS results. In cohort A, 5/11 (45%) patients with a targetable mutation (15% of the whole cohort A) received targeted therapy, whereas in cohort B, 3/16 (19%) patients with a targetable mutation (4.5% of the whole cohort B) received targeted therapy (Table 2 and Table 3 and Figure 8). The differences between the cohorts were not statistically significant (*p* = 0.2).

We found the following reasons why in our collective only 45% of patients with targetable mutations in cohort A and 19% in cohort B started targeted therapy and why older patients (cohort B) were less likely to receive targeted therapy:

Missing follow-up data (1 patient cohort A, 2 patients cohort B), unknown (3 patients cohort A, 1 patient cohort B), rapidly progressive disease/death (1 patient cohort A and 1 patient in cohort B), and stable disease under prior therapy (1 patient cohort A and 2 patients in cohort B). In cohort B, additional reasons were no therapy request (2 patients), reduced general condition (2 patients), comorbidities (1 patient), and status after targeted therapy has taken place (2 patients).

In total, 3/8 patients (38%) with a targeted therapy (3% of the study cohort) benefited from the therapy (PFS > 6 months; cohort A, n = 1; cohort B, n = 2). As follow-up for one patient from cohort B was unavailable, it is unclear whether he received targeted therapy. In cohort A, no patient had complete remission under targeted therapy, one patient had stable disease, three patients had progressive disease, and one patient had to discontinue medication due to toxicity. In cohort B, two patients had a partial response, and one patient had progressive disease within 16 weeks of targeted therapy (Figure 8). Overall, 67% of patients with a targetable mutation did not receive targeted therapy.

**Figure 9:**
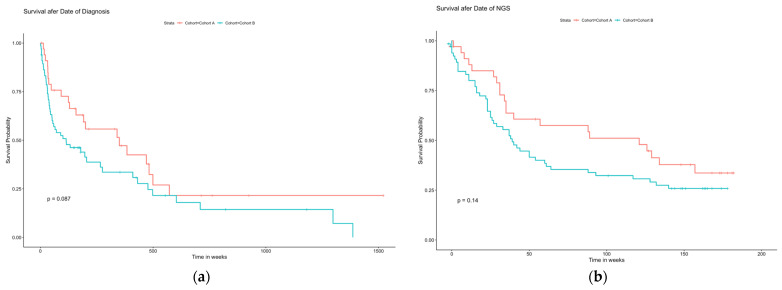
Survival analysis. (**a**) After the date of diagnosis. (**b**) After the date of NGS.

Overall survival analysis showed no statistically significant differences between cohorts A and B in survival after the date of diagnosis and survival after the date of NGS (Figure 9). There was a trend toward better OS in younger patients after the date of diagnosis, with a median overall OS of 150 (1–1521) weeks; the median OS was 192 (4–1521) weeks in cohort A and 197 (1–1429) weeks in cohort B (*p* = 0.08).

#### Overall Survival Analysis

**Figure 10:**
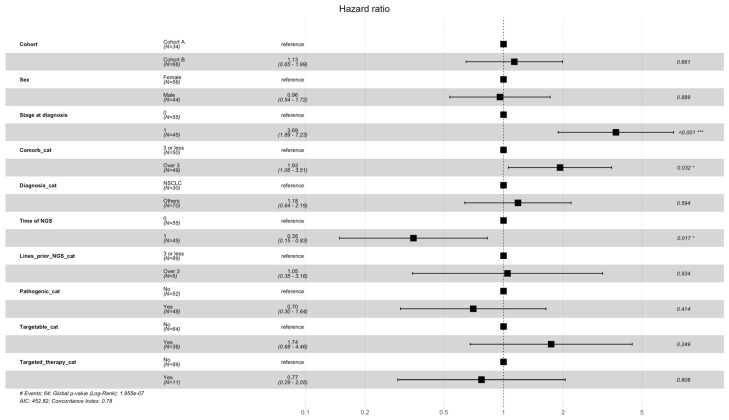
Hazard ratios for overall survival. OS was stratified by different subgroups, including cohort A/B, Sex, Stage, Comorbidities, Diagnosis, Time of NGS, Lines prior to NGS, Pathogenic categories, Targetable yes/no, and receiving Targetable agents. * = statistically significant, *** = statistically highly significant.

The hazard ratio calculations (Figure 10) showed that the number of comorbidities (≥3, HR 1.88; 95% CI 1.01–3.52; *p* = 0.048) and a metastatic stage at diagnosis (HR 3.75; 95% CI 1.88–7.47; *p* < 0.001) had statistically significant negative effects on survival. A later NGS analysis (not at diagnosis but later during the disease course) was associated with a statistically significant positive effect on survival (OR 0.35; 95% CI 0.15–0.82; *p* = 0.015).

## 3. Materials and Methods

This retrospective study was conducted at the Cancer Center Baselland, Liestal, Switzerland, which is the cancer unit of the Hospital of Canton Baselland (KSBL). We assessed demographic, clinical, pathological, and disease-specific data from 100 patients with cancer who underwent NGS at our institution between January and December 2020. This study aimed to improve the understanding of the use and utility of NGS data in older patients with cancer. Therefore, we compared the clinicopathologic and NGS data of 100 randomly selected patients with solid cancer of any subtype and any TMN stage who underwent NGS testing in 2020 at our institution and correlated the results with the outcomes in the non-older (A: age < 70 years) and older (B: age ≥ 70 years) cohorts of patients with cancer in a real-world setting.

### 3.1. Objectives

1. To compare the clinicopathologic and NGS data in the two cohorts (A and B)
2. To compare the rate of presentation to the molecular tumor board and discussion with experts
3. To compare the outcomes of patients in cohorts A and B based on the successful allocation of NGS-data-based precision therapy (time of treatment)

### 3.2. Endpoints

1. Clinical characteristics in descriptive statistics in cohorts A and B
2. Rate of molecular tumor board presentation (optimal 1 NGS = 1 tumor board presentation in cohorts A and B)
3. Time on treatment with the targeted agent after the NGS-based treatment allocation

### 3.3. Patients Disposition and Cohort Description

Patient selection was based on a report prepared by the Institute of Pathology of our hospital, in which all patients with tumors who underwent an NGS examination in 2020 were listed in random order. The selection criteria for the patients are presented in Appendix A.

The first 100 consecutive patients were included in this study. All clinicodemographic patient information was collected from the KSBL’s electronic medical records/charts (see Appendix B for detailed information) and transferred to an MS Excel^®^ file (https://www.office.com/).

In the next step, all information on the NGS analyses was compiled from electronic pathology reports (see Appendix C). All patients who were under 70 years old at the time of NGS analysis were randomized in cohort A; all patients who were 70 years or older at the time of NGS analysis were randomized in cohort B. All mutations and amplifications identified by NGS were recorded, and the predictive/pathogenic mutations according to the pathology report were noted. The latest (data: 24 January 2024) OnkoKB^®^ [23] database was used to determine whether a target or targeted therapy was available for these predictive/pathogenic mutations. Patient files were then searched to determine whether the NGS data had been discussed at a molecular tumor conference and if the patients for whom the targeted therapy was available had received it. Further information about targeted therapy, such as the name of therapy, time on therapy, best response, progression-free survival (PFS), benefit of therapy (PFS > 6 months), and time of overall survival (OS), was noted. Finally, the date of last follow-up or death was recorded. Patients who received NGS-based treatment were classified according to the current ESMO (European Society for Medical Oncology) ESCAT (ESMO Scale for Clinical Actionability of Molecular Targets) criteria [24].

### 3.4. Molecular Diagnostics

All NGS analyses were performed at the Institute of Pathology of our hospital using nationally certified tests. Ninety-seven percent of the analyses were performed on FFPE (formalin-fixed paraffin-embedded) tissues. Three analyses were performed on the liquid biopsies. The assays were performed using local standard operating procedures according to the manufacturer’s guidelines.

The following NGS panels were used (see Appendix D for detailed information about the NGS panels and a list of the whole genes that were examined).

(A) Oncomine Comprehensive Assay^®^, Version 3 (OCAv3) (ThermoFisher Scientific, Waltham, MA, USA): a multi-biomarker NGS assay that covers 161 of the most relevant cancer genes
(B) Oncomine Tumor Mutation Load Assay^®^ (TML) (ThermoFisher Scientific, Waltham, MA, USA): a large targeted NGS assay that covers 1.65 Mb across 409 oncogenes relevant across major cancer types
(C) Oncomine Solid Tumor DNA Panel^®^ (ThermoFisher Scientific, Waltham, MA, USA): next-generation sequencing (NGS) kits for both DNA and RNA workflows that enable the assessment of actionable mutations involved in solid tumors, particularly those originating from the lung and colon, and cover 22 hotspot genes
(D) KSBL Hotspot Panel^®^ (HOP), (Institute of Pathology of Liestal, Switzerland): The KSBL Hotspot Panel is a custom panel designed by the Institute of Pathology of Liestal, based on the Ion Ampliseq Custom DNA panels (Thermo Fisher Scientific, Waltham, MA, USA). It partially covers 124 genes for hotspot alterations and 34 genes for copy number variations.
(E) Ion Torrent™ Oncomine™ cfDNA Assays (ThermoFischer Scientific, Waltham, MA, USA): tumor type-specific multi-biomarker next-generation sequencing (NGS) assays that enable the detection of somatic mutations in genes found in plasma samples
  - Oncomine Lung cfDNA Assay^®^ covers 11 hotspot genes
  - Oncomine Colon cfDNA Assay^®^ (cfColon) covers 14 hotspot genes

Data readout and analyses were performed by an accredited molecular pathologist at the Institute of Pathology.

### 3.5. Statistical Analysis

All data were analyzed by a statistician at our hospital. A comprehensive set of statistical and analytical methods was employed to elucidate the key aspects of the dataset. Using the R programming language (The R Foundation for Statistical Computing, c/o Institute for Statistics and Mathematics, University of Economics and Business Vienna, Austria), various functions and techniques were applied to derive meaningful insights.

The following statistical methods were utilized:

1. Data Exploration and Grouping:
  ○ The count and group functions in R (The R Foundation for Statistical Computing c/o Institute for Statistics and Mathematics, University of Economics and Business Vienna, Austria) were used to systematically examine and categorize cases based on specific criteria, enabling a detailed exploration of the dataset.
2. Descriptive Statistics:
  ○ To calculate the median, minimum, and maximum values for selected variables to provide a summary of the central tendency and variability of the data.
3. Statistical Testing:
  ○ Applied the two-sided Mann–Whitney *U* test to assess the significance of differences between the median values for two distinct groups. This method provides a robust nonparametric approach for identifying potential contrasts in central tendencies.
4. Survival Analysis: (survival package)
  ○ Implemented the surv() function in R to create survival objects that define survival times and event indicators for each participant.
  ○ The survfit () function was used to compute the Kaplan–Meier survival curves, which visually represent the probability of survival over time.
5. Cox Proportional Hazards Model: (survival package)
  ○ The coxph () function for the Cox proportional hazards model was used to calculate the hazard ratios and their associated confidence intervals.
  ○ Generated a forest plot using the forestplot package to visualize the results.
6. Plotting:
  ○ Plots were generated using the ggplot2 package in R (https://ggplot2.tidyverse.org/), which provides clear and visually informative representations of various analyses, including descriptive statistics, survival curves, and forest plots.

### 3.6. Ethical Approval

This study was approved by the local ethics committee, Ethikkomission Norwest-und Zentralschweiz (EKNZ; approval no. BASEC-Nr. 2023-00602). The need for informed consent was waived by EKNZ.

## 4. Discussion

This retrospective single-center study aimed to analyze the clinicopathologic, molecular, and treatment data of 100 patients who underwent NGS in 2020 at our institution. The study compared two cohorts: cohort A comprised patients younger than 70 years at the time of NGS, and cohort B comprised patients aged 70 years or older at the time of NGS.

### 4.1. Clinical Differences of the Cohorts and Differences in NGS

Despite random patient selection, our cohort was not well-balanced, with one-third patients in cohort A and two-thirds in cohort B. This contradicts our hypothesis that NGS is used more frequently in younger patients with cancer. However, this reflects that cancer is a disease of older patients, and NGS is currently a standard diagnostic tool that is used in many different tumor entities, for example, lung cancer, colorectal cancer, prostate cancer, and breast cancer, depending on the approval status of the targeted agents [25].

The number of comorbidities increased with age, and the difference between the two cohorts was statistically significant. This finding aligns with the current literature and should be considered when performing NGS testing [26]. Benderra et al. demonstrated that, in their ELCAPA cohort of more than 1500 older patients with cancer, the comorbidity type, number, and severity were independently associated with a shorter OS. However, the number of comorbidities should not be a barrier to testing older patients with cancer. This is because NGS-driven personalized treatment approaches might increase the tolerability and quality of life of older patients with cancer [27].

Considering the results of the ECOF PS, one can assume that our patient population was quite fit at the time of NGS analysis, with 80% having a documented ECOG PS between 0 and 1 and 20% with an ECOG PS of 2–3. No patient had an ECOG PS of 4, indicating that patients who were completely disabled (ECOG PS 4) were not the target audience for NGS analysis. Other studies have shown that NGS is not valuable when performed in cases with a poor performance status [28]. In general, it is important to question whether the ECOG PS is the correct screening tool for older patients to determine a therapeutic strategy. The ECOC PS describes only the patient’s level of functioning in terms of their ability to care for themselves, daily activity, and physical ability and does not account for the heterogeneity among older adults with respect to their biological age [29,30]. There are special geriatric assessment tools that refer to the evaluation of geriatric impairments that are not routinely captured in oncology assessments by evaluating the functional, cognitive, psychological, and nutritional status, physical performance, history of falls, comorbid medical conditions, and social support, which helps to estimate the biological age [31]. As we aimed to assess the data of the G8 questionnaire (a screening tool that was developed to identify older patients with cancer who would benefit from a former comprehensive geriatric assessment) and correlate with our findings [32]. However, systematic data collection has only been implemented at our cancer center since 2024.

Thus, the clinical management of older patients with cancer should follow the rules of the current guidelines and include a full geriatric assessment if the results of screening procedures, such as the G8 Score, are positive [33,34]. Treatment decisions based on NGS testing might be better facilitated by a more personalized geriatric oncology approach. A personalized geriatric oncology approach for older cancer patients can enhance treatment decisions based on NGS testing by tailoring interventions to their unique health profiles. This includes using comprehensive geriatric assessments (CGA) to adjust treatment intensity, prioritizing targeted therapies with fewer side effects, setting individualized treatment goals focused on quality of life, and applying pharmacogenomic data to reduce toxicity and manage polypharmacy. These strategies help clinicians balance effective cancer treatment with the complex needs of older adults, improving both outcomes and quality of life.

Our patient cohort comprised patients with a heterogeneous group of cancers. We observed a relatively high proportion of patients with newly diagnosed advanced NSCLC, colorectal cancer, and melanoma in cohort B, in which NGS was the standard of care at primary diagnosis [35,36,37]. This suggests that lung cancer is the most common newly diagnosed cancer in patients older than 70 years, followed by colorectal, prostate, and breast cancer [38]. In our study, we observed a relatively low rate of prostate cancer.

It is possible that the more frequent occurrence of one tumor entity may have influenced the results for other tumor entities. However, the most frequently diagnosed tumor entities are also frequently represented in our study population, which also corresponds to the real-world setting.

Patients in cohort A were more frequently diagnosed with localized cancer (65%) than those in cohort B, of whom 50% had metastatic cancer. This indicates that older patients with cancer are often diagnosed at later stages. However, we estimated that our cohort was too small to draw any conclusions on this topic.

### 4.2. Number of Treatment Lines in Metastatic Cancers

Patients in cohort B received a significantly lower total number of treatment lines than those from cohort A (median number of 6 treatment lines in cohort A vs. 2 in cohort B). Based on current literature, there is a risk of undertreatment in older patients. Older patients are more likely to be frail; therefore, treatment decisions may be more conservative [39,40,41]. A large proportion of older patients also have rapidly progressive disease, refuse therapy, and die rapidly. Only a minority of patients received more than three lines prior to NGS testing (three patients each from cohorts A and B; total 6%).

### 4.3. Results of NGS Analysis

NGS was performed in 55% at diagnosis and in 45% at a later timepoint. In cohort B, NGS analysis was more common at diagnosis than in cohort A (62% vs. 41%, *p* = 0.0747). Based on our selected NGS panels, common cancer mutations were detected in both cohorts, including tp53 (40%), KRAS (24%), SETD2 (12%), SLX4 (12%), and ATR (11%) (Figure 2 and Figure 3). *TP53* was the most commonly mutated gene, and the number of *KRAS* mutations in our study was higher than that reported in other studies [42].

The proportion of patients with pathogenic mutations was similar in the two cohorts (47% in cohort A and 48.5% in cohort B). The proportion of patients with targetable mutations was numerically higher in cohort A than in cohort B (32.5% vs. 24%); this difference was not statistically significant (*p* = 0.476). This demonstrates that older patients with cancer might have a different biology of their disease; this has been shown in several publications [43]. This result limits the treatment options with targeted therapy in older patients. However, the cohort is too small and heterogeneous to draw general conclusions from it.

### 4.4. Outcome After NGS

In total (both cohorts), 27% of patients had targetable mutations, and 8% of our patients received targeted therapy. In cohort A, 5/11 (45%) patients with a targetable mutation (15% of the whole cohort A) received targeted therapy, whereas in cohort B, 3/16 (19%) patients with a targetable mutation (4.5% of the whole cohort B) received targeted therapy. Although the difference between 45% treatment initiation in cohort A versus 19% in cohort B was not statistically significant, these figures show that targeted therapy was started less frequently in older cancer patients. It is possible that the low number of cases in both cohorts influenced the statistical significance. However, we think that the clinical importance of this result is relevant for older patients because they were less likely to be treated with targeted therapy tailored to their tumor mutation.

But compared to other cohorts, this was in general a comparable outcome for our patients. A study from 2020, which analyzed the impact of NGS on clinical practice in oncology in France, analyzed NGS data from 2013 to 2016 and found actionable alterations in 53% of which 8% received targeted therapy finally [44]. They reported also from studies with sample sizes ranging from 97 to 17 664 patients, with the percentage of patients with actionable alterations ranging from 40% to 94% and of patients receiving matched treatment ranging from 4% to 44% [45]. In the analysis from Gibbs et al. (2023), who performed a comprehensive literature review on the clinical impact of NGS for the management of advanced cancer, they found a mean of 29% and a median of 25% (range 2–66%) of patients that were matched to targeted treatment after NGS testing [46].

Some reasons why less than 50% of the patients with targetable mutations in cohort A (5/11 patients, 45%) and only 19% from cohort B (3/16) started targeted therapy include rapidly progressive disease and stable disease under prior therapy for both cohorts. In cohort B, also age-specific factors such as reduced general condition, no therapy request, and comorbidities, as well as status after targeted therapy, were reasons to not start a targeted therapy. Similar reasons are mentioned in other studies [45,46,47].

Of the 8% who started targeted therapy, 3 patients (38%) or 3% of the whole cohort experienced a clinical benefit from the targeted therapy. One patient from cohort A with NSLCL treated with lorlatinib was treated for at least 168 weeks, with ongoing therapy and stable disease. Two patients from cohort B had partial remission; one patient with GIST was treated with imatinib for 104 weeks, and the other patient with breast cancer who was treated with fulvestrant for at least 136 weeks had ongoing therapy. For those three patients, targeted therapy changed the way of treatment for the last years, and according to the medical history, they are all in a good clinical condition due to the precision medicine.

The reasons for the other five patients without clinical benefits were progressive disease in four patients and toxicity-related discontinuation of therapy in one patient.

We did not find a difference in the clinical outcomes after targeted therapy between cohorts A and B because of the very small number of patients. However, we highlight that a small but relevant portion of older patients benefited from NGS-determined therapies. In addition, despite the lower uptake of targeted therapies among older patients, the positive outcome for those who did receive them suggests a need to reconsider current clinical approaches to reduce the risk of undertreatment of older cancer patients.

Our result of the benefit of targeted therapy after NGS testing of 3% for the whole cohort is consistent with published data. Marquart et al. reported in an American observational study that 8.3% of patients received targeted therapy and 4.9% benefited from a genome-targeted treatment [14]. In the MOSCATO Trial, they reported a rate of 19% of patients matched to targeted therapy and a clinical benefit of 33% of these patients, or 7% of the whole cohort [15]. Another study, part of the TAPUR (Targeted Agent and Profiling Utilization Registry) and NCI-MATCH (Molecular Analysis for Therapy Choice) trials, analyzed the impact of NGS on therapy decisions in patients with metastatic cancer. The study found that NGS testing led to treatment changes in approximately 16% of cases, with a notable proportion of these patients experiencing significant clinical benefits from targeted therapies identified through NGS. The study highlighted that while NGS can reveal actionable mutations, the benefit varies, with some patients showing exceptional responses to therapies tailored to their genetic profile [16,17]. We could not find any other trails, that examined the effectiveness of NGS results in relation to patient age.

Limiting treatment success includes late testing, poor prognosis, therapy-refractory disease, and patient death.

Due to the small number of patients and, in some cases, only a few patients with the same tumor diseases, comparative cancer subgroup analyses, i.e., to analyze cancer-specific trends in the utility or success of NGS-based therapies, were not possible. We found targetable mutations in patients with NSCLC, breast cancer, colorectal cancer, melanoma, sarcoma, GIST, and CUP using NGS examination, and targeted therapies were started in patients with NSCLC (2 patients from cohort A), breast cancer (1 patient from cohort A and 2 patients from cohort B), colorectal cancer (1 patient from cohort A), sarcoma (1 patient from cohort A), and GIST (1 patient from cohort B). From cohort A, one patient with NSCLC, and from cohort B, one patient with breast cancer, and one patient with GIST have benefited from the NGS-guided treatment. However, it is important to note that this result is the result of our cohort and cannot be considered as generally valid because many individual factors led to no therapy being started for other cancer subtypes in both cohort A and cohort B. In cohort B, for example, 4 patients with melanoma had targetable mutations, and all patients were refused the start of targeted therapy (due to bad general condition, no therapy request, loss of FU, and therapy not started yet). It could be that in another cohort all patients are started on therapy, which would lead to different results than in our collective. Therefore, larger data collections are necessary to confirm our results.

### 4.5. Overall Survival

After NGS, the prognosis was generally very poor, with a median OS of less than 12 months in both cohorts. There was no statistically significant difference in OS between cohorts A and B, either after the date of diagnosis or after the date of NGS, with a trend toward better overall survival in younger patients after the date of diagnosis (*p* = 0.08).

One reason why younger patients did not have better overall survival than older patients could be that cancer death is related to treatment, treatment duration, and number of lines. We observed that our patients from cohort A were treated with a significantly higher number of therapy lines and had a higher cancer burden and more refractory disease than older patients. On the other hand, a non-significant numerical difference was observed in older patients. Older patients with cancer have a higher competing risk to die because of comorbidities and side effects of cancer therapy. Nevertheless, questions remain, and one might hypothesize about the factors influencing overall survival (OS), including decision-making based on NGS results in older cancer patients. Further research is needed to clarify these factors, and more studies are needed to confirm our observations and achieve significant results.

Factors that had a statistically significant negative effect on overall survival were a higher number of comorbidities at diagnosis (>3) and metastatic stage at diagnosis.

The administration of targeted therapy had no statistically significant effect on OS in our cohort. We attribute this to the small number of patients who received targeted therapy. Another large retrospective cohort study conducted at the Institut Curie in France assessed the survival impact of targeted therapies in lung cancer patients over two decades. The study found that the introduction of targeted therapies, guided by NGS, significantly improved overall survival (OS) in patients with non-small cell lung cancer (NSCLC). The median OS increased notably following the adoption of these therapies, highlighting the clinical benefit of NGS-driven treatment strategies in this specific population [18].

### 4.6. Discussion of Results at Molecular Tumor Board/Tumor Board

Our second endpoint was to evaluate the number of NGS results presented and discussed on the molecular tumor board. At our institution, we joined the molecular tumor board together with the University Hospital of Basel. In 2020, the results of NGS analyses were discussed at the molecular tumor board for only a minority of patients. Most of our patients were discussed with our institutional tumor board, where the decision to perform the NGS analysis was made. In only 14% of our patients (18% of patients in cohort A and 12% of patients in cohort B), the results of the NGS analysis were discussed again by the tumor board. On the one hand, this is due to the fact that we had many patients in whom no predictive mutations were found (53%). However, data from pathology reports are used in routine clinical practice, and common mutations such as EGFR in lung cancer and ESR1 or PIK3CA mutations in breast or colon cancer are not present on the molecular board. Furthermore, in 2020, the majority of patients with molecular alterations in early disease did not receive any molecular-driven treatment and were therefore not regularly presented on the molecular tumor board.

### 4.7. Limitations

The limitations of our data include the small sample size, single center data, hypothesis generating data, retrospective nature, and heterogeneous tumor entities.

### 4.8. Potential Biases

One potential bias could be the different tumor stages at the time of NGS. Fifty-nine percent of patients of cohort A had NGS not at the timepoint of diagnosis but later in the course of disease, while 62% of the patients of cohort B had the NGS examination at the timepoint of diagnosis. This maybe has influenced the decision for treatment (although we could not prove this with our data because of the lack of information about the reasons for not starting targeted therapy for many patients from cohort A). Another weakness of our analysis might be the bias in terms of OS/PFS analysis due to the high number of comorbidities in both cohorts, which could have influenced our OS and PFS results. However, due to the random selection of patients, we have reduced biases and present a Swiss real-world cohort.

### 4.9. Strengths

We compared the data from the two cohorts in the real-world setting without exclusion of patients due to older age or comorbidities.

## 5. Conclusions

In this small retrospective single-center analysis, we were able to characterize the clinical and molecular differences between younger and older patients with cancer and showed that the diagnostic approach and treatment strategy did not differ with regard to age. A small but relevant proportion of patients (3%) benefited from the results of NGS analyses. For these three patients, targeted therapy changed the way of treatment. They had a significant treatment benefit with a time on treatment of 104 to at least 168 weeks with ongoing therapy for two of the three patients. Nevertheless, older patients with cancer who had targetable mutations in our study received targeted therapy less frequently than younger patients, although this difference was not statistically significant.

To confirm our results, we are planning to continue our registry as a prospective registry of older cancer patients and also analyze the NGS analyses conducted by our institution in recent years. This would also allow us to make different specific subgroup analyses (i.e., cancer-type specific subgroup analyses).

There is a dire need to include older patients with cancer in new precision medicine trial protocols to obtain prospective clinical data about the role and impact of NGS in older cancer patients. It is very important that further research in precision medicine does not exclude older cancer patients due to age or comorbidities, so that treatment options are evaluated for patients in a real-world setting. We are convinced that NGS analyses will improve the outcome of tumor patients in all age groups, provided that the current diagnostic and treatment recommendations are considered and an increasingly multidisciplinary approach to the diagnosis and treatment of tumor patients is adopted.

## Figures and Tables

**Table 1:** Patient characteristics.

	Overall, N (%)	Cohort A (<70 y) N (%)	Cohort B (≥70 y) N (%)	Level of Significance
Number of patients (%)	100	34	66	
Age				
Age at primary diagnosis, median (range)	71 (29–89)	56 (29–69)	74 (48–89)	
Age at relapse, median (range)	68 (29–84)	57 (29–69)	74 (64–84)	
Age at NGS, median (range)	72 (35–89)	59 (35–69)	76 (70–89)	
Sex				
Females	56	19 (56%)	37 (56%)	
Males	44	15 (44%)	29 (44%)	
Number of comorbidities at diagnosis, median (range)	3 (0–10)	2 (0–10)	4 (0–10)	Mann–Whitney-*U* test, two-sided, W = 779.5, *p* = 0.01583
Number of patients with the following comorbidities				
Cardiovascular	62	20 (59%)	42 (63.5%)	
Pulmonal	24	8 (23.5%)	16 (24%)	
Diabetes	21	6 (17.5%)	15 (23%)	
Renal insufficiency	13	2 (6%)	11 (16.5%)	
ECOG Performance Status at Diagnosis				
0	19	7 (20.5%)	12 (18%)	
0–1	4	1 (3%)	3 (4.5%)	
1	32	10 (29.5%)	22 (33%)	
2	10	2 (6%)	8 (12%)	
3	6	3 (9%)	3 (4.5%)	
4	0	0	0	
Unknown	29	11 (32%)	18 (27%)	
Diagnosis				
1: NSCLC	1: 30	1: 6 (18%)	1: 24 (36%)	
2: SCLC	2: 1	2: 1 (3%)	2: 0	
3: Breast cancer	3: 13	3: 6 (18%)	3: 7 (11%)	
4: Colorectal cancer	4: 16	4: 9 (26%)	4: 7 (11%)	
5: Prostate cancer	5: 4	5: 1 (3%)	5: 3 (4.55%)	
6. Low-grade lymphoma	6: 2	6: 0	6: 2 (3%)	
7: Pancreatic cancer	7: 1	7: 0	7: 1 (1.5%)	
8: Mesothelioma	8: 1	8: 0	8: 1 (1.5%)	
9: Sarcoma	9: 3	9: 1 (3%)	9: 2 (3%)	
10: Endometrial cancer	10: 1	10: 0	10: 1 (1.5%)	
11: Ovarian cancer	11: 5	11: 2 (6%)	11: 3 (4.55%)	
12: Esophageal cancer	12: 2	12: 1 (3%)	12: 1 (1.5%)	
13: GIST	13: 2	13: 0	13: 2 (3%)	
14: Melanoma	14: 9	14: 2 (6%)	14: 7 (11%)	
15: Uterine tube cancer	15: 1	15: 0	15: 1 (1.5%)	
16: Kidney cancer	16: 1	16: 1 (3%)	16: 0	
17: CUP	17: 4	17: 2 (6%)	17: 2 (3%)	
18: Urothelial cancer	18: 2	18: 1 (3%)	18: 1 (1.5%)	
19: Cholangiocellular cancer (CCC)	19: 2	19: 1 (3%)	19: 1 (1.5%)	
Stage at diagnosis				
Local	55	22 (65%)	33 (50%)	
Metastatic	45	12 (35%)	33 (50%)	
Treatment				Mann–Whitney-*U* test, two-sided, W = 1105, *p* = 0.001324
Number of lines, median (range)	2 (1–15)	6 (1–12)	2 (1–15)	
Number of lines before NGS, median (range)	0 (0–11)	0 (0–7)	0 (0–11)	
Number of lines	0: 65 1: 17 2: 3 3: 5 4: 2 5: 2 7: 1 11: 1	0: 18 (53%) 1: 7 (21%) 2: 3 (9%) 3: 3 (9%) 5: 2 (6%) 7: 1 (3%)	0: 47 (71%) 1: 10 (15%) 3: 2 (3%) 4: 2 (3%) 11: 1 (1.5%) Unknown: 4	

**Table 2:** Results of NGS.

	Overall, N (%)	Cohort A (<70 y), N (%)	Cohort B (≥70 y), N (%)	
Time of NGS in 2020				Pearson’s chi-square test with Yates’ continuity correction
At diagnosis	55	14 (41%)	41 (62%)	data: time_ngs_table
Later/in the disease course	45	20 (59%)	25 (38%)	X-squared = 3.1761, df = 1, *p* = 0.07472
NGS Panel used				
1: Oncomine Comprehensive Assay^®^, Version 3 (OCAv3)	87	28 (82%)	59 (89%)	
2: Oncomine Tumor Mutation Load Assay^®^ (TML)	1	1 (3%)	0	
3: Oncomine Solid Tumor Panel^®^	1	0	1 (1.5%)	
4: KSBL Hotspot Panel^®^ (HOP)	2	1 (3%)	1 (1.5%)	
5: Oncomine Lung cfDNA Assay^®^	2	1 (3%)	1 (1.5%)	
6: Oncomine Colon cfDNA Assay^®^ (cfColon)	2	2 (6%)	0	
7: Unknown	5	1 (3%)	4 (6%)	
Number of detected mutations	109	71	94	
Pathogenic mutations				
Number of patients with 0–3 pathogenic mutations	0: 52 1: 38 2: 9 3: 1	0: 18 (53%) 1: 12 (35%) 2: 4 (12%)	0: 34 (51.5%) 1: 26 (39%) 2: 5 (8%) 3: 1 (1.5%)	
Median number of pathogenic mutations (range) per patient	0 (0–3)	0 (0–2)	0 (0–3)	
Total number of patients with pathogenic mutations	48	16 (47%)	32 (48.5%)	
Targetable mutations				
Number of patients with 0–3 targetable mutations	0: 73 1: 22 2: 4 3: 1	0: 23 (67.5%) 1: 8 (23.5%) 2: 3 (9%)	0: 50 (76%) 1: 14 (21%) 2: 1 (1.5% 3: 1 (1.5)	*p* = 0.4767 Alternative hypothesis: true odds ratio is not equal to 1 95% confidence interval: 0.5342746–4.0636835 Sample estimates: odds ratio: 1.488441
Total number of patients with targetable mutations	27	11 (32.5%)	16 (24%)	
Number of NGS results discussed at the tumor board				
No	86	28 (82%)	58 (88%)	
Yes	14	6 (18)	8 (12%)	
Reasons why the NGS results were not discussed at the tumor board				
1: No therapy-relevant mutation	53	17 (50%)	36 (55%)	
2: No therapy requests	3	0	3 (5%)	
3: Unknown	14	6 (18%)	8 (12%)	
4: Stable under previous/actual line	3	3 (9%)	0	
5: Death shortly after NGS	7	0	7 (10%)	
6: Treatment at another institution	6	2 (6%)	4 (6%)	
Patients who started targeted therapy	Of the 27 patients with a targetable mutation:	Of the 11 patients with a targetable mutation:	Of the 16 patients with a targetable mutation:	Fischer’s test for difference: *p* = 0.2064
No	18 (67%)	6 (55%)	12 (75%)	Alternative hypothesis: true odds ratio is not equal to 1
Yes	8 (30%)	5 (45%)	3 (19%)	95% confidence interval: 0.4809042–30.0408206
Unknown	1 (3%)	0	1 (6%)	Sample estimates: odds ratio: 3.429212
Time on treatment with targeted therapy after allocation to NGS-based treatment (weeks)	11 w: 1 16 w: 1 16 w+ unknown: 1 26 w: 1 8 w: 2 104 w: 1 Ongoing: 2	11 w: 1 16 w+ unknown: 1 8 w: 2 Ongoing: 1	16 w: 1 26 w: 1 104 w: 1 Ongoing: 1	
Patients with clinical benefit of targeted therapy (at least 6 months on therapy without progression)				
No clinical benefit	5	4	1	
Clinical benefit	3	1	2	
Reason why targeted therapy was not started				
1: Not started yet/stable disease under previous therapy	3	1	2	
2: No therapy request	2	0	2	
3: Poor general condition	2	0	2	
4: Progressive disease/death	2	1	1	
5: Unknown	4	3	1	
6: Lost to follow-up	3	1	2	
7: Prior targeted therapy	2	0	2	
8: Contraindication (e.g., proteinuria)	1	0	1	
Time from NGS to death independent of therapy, weeks, median (range)	33.4 (−2.7 to 157)	35.3 (7.3–157)	26.7 (−2.7 to 132)	
Status at the end of the data-collection period				
Alive	31	13 (38%)	18 (27%)	
Dead	61	19 (56%)	42 (64%	
Lost to follow-up	8	2 (6%)	6 (9%)	
Survival since diagnosis, weeks, median (range)	150 (1–1521)	192 (4–1521)	197 (1–1429)	

**Table 3:** Clinical information of patients who received targeted therapy.

Sex	Age (Years) at NGS	Diagnosis	Time of NGS (at Diagnosis or Later)	Targetable Mutation	Targeted Therapy	ESCAT Level TIERs	Time on Treatment (Weeks)	Best Response	Progression-Free Survival	Clinical Benefit
f	73	Breast cancer	Later	*ESR1*	Fulvestrant	IA	Ongoing (mind 136)	PR	Mind 136	Yes
	59	Sarcoma	Later	*CDK4*	Palbociclib	IV	8	Stopped because of toxicity	8	No
f	63	NSCLC	Later	*ALK*	Lorlatinib	IA	Ongoing (mind 168)	SD	Mind 168	Yes
f	62	NSCLC	Diagnosis	*BRAF (pV600E)*	Dabrafenib+ Trametinib	IA	11	PD	11	No
f	57	Breast cancer	Later	*PIK3CA*	Alpesilib	IA	8	PD	8	No
m	70	GIST	Diagnosis	*KIT*	Imatinib	IA	Mind 104	PR	Mind 104	Yes
f	72	Breast cancer	Later	*ESR1*	Fulvestrant + Palbociclib	IA	16	PD	16	No
m	35	Colorectal cancer	Later	*BRAF (pV600E)*, *MAP2K1*	Dabrafenib + Trametinib	IA	16 + unknown	PD	16+ unknown	No

PR = partial remission, SD = stable disease, PD = progressive disease.

## Data Availability

The data presented in this study are available on request from the corresponding author due to privacy reasons.

## Appendix A. Selection Criteria for Inclusion and Exclusion in the Study

Inclusion criteria: Adults (age ≥ 18 years) diagnosed with cancer who underwent NGS testing at our institution in 2020.

Exclusion criteria: No evidence of a histological diagnosis of cancer or missing NGS data, and those with recorded written or verbal denial of consent for clinical procedures and NGS data.

## Appendix B. Demographics and Clinical Information of Patients Obtained from the KSBL Electronic Medical Records/Charts

Sex, date of birth, date of diagnosis, number of comorbidities at diagnosis, G8 status, ECOG performance status at diagnosis, diagnosis, stage at diagnosis, tumor board presentation at diagnosis, neoadjuvant and adjuvant treatments, date and type of relapse, age at relapse, metastatic sites, and palliative treatment.

## Appendix C. Information on the NGS Analyses Compiled from the Electronic Pathology Reports

Date of NGS, age at NGS, timepoint of NGS (at diagnosis or later), number of therapy lines prior to NGS, and type of NGS panel used.

## Appendix D. NGS Panels Used and Genes Examined in the Different NGS Panels

(A) Oncomine Comprehensive Assay^®^, Version 3 (OCAv3) (ThermoFisher Scientific, Waltham, MA, USA):

A multi-biomarker NGS assay that covered 161 of the most relevant cancer genes, based on the Oncomine Knowledgebase and collaborations with leading cancer research institutions and pharmaceutical companies. The low sample-input requirement enables the analysis of even small and challenging formalin-fixed paraffin-embedded (FFPE) samples.

86 Hotspot genes*: AKT1 AKT2 AKT3 ALK AR ARAF AXL BRAF BTK CBL CCND1 CDK4 CDK6 CHEK2 CSF1R CTNNB1 DDR2 EGFR ERBB2 ERBB3 ERBB4 ERCC2 ESR1 EZH2 FGFR1 FGFR2 FGFR3 FGFR4 FLT3 FOXL2 GATA2 GNA11 GNAQ GNAS H3F3A HIST1H3B HNF1A HRAS IDH1 IDH2 JAK1 JAK2 JAK3 KDR KIT KNSTRN KRAS MAGOH MAP2K1 MAP2K2 MAP2K4 MAPK1 MAX MDM4 MED12 MET MTOR MYC MYCN MYD88 NFE2L2 NRAS NTRK1 NTRK2 NTRK3 PDGFRA PDGFRB PIK3CB PIK3CA PPP2R1A PTPN11 RAC1 RAF1 RET RHEB RHOA ROS1 SF3B1 SMAD4 SMO SPOP SRC STAT3 TERT TOP1 U2AF1 XPO1*

48 Full-length genes: *ARID1A ATM ATR ATRX BAP1 BRCA1 BRCA2 CDK12 CDKN1B CDKN2A CDKN2B CHEK1 CREBBP FANCA FANCD2 FANCI FBXW7 MLH1 MRE11 MSH6 MSH2 NBN NF1 NF2 NOTCH1 NOTCH2 NOTCH3 PALB2 PIK3R1 PMS2 POLE PTCH1 PTEN RAD50 RAD51 RAD51B RAD51C RAD51D RNF43 RB1 SETD2 SLX4 SMARCA4 SMARCB1 STK11 TP53 TSC1 TSC2*

43 Copy number genes: *AKT1 AKT2 AKT3 ALK AXL AR BRAF CCND1 CCND2 CCND3 CCNE1 CDK2 CDK4 CDK6 EGFR ERBB2 ESR1 FGF19 FGF3 FGFR1 FGFR2 FGFR3 FGFR4 FLT3 IGF1R KIT KRAS MDM2 MDM4 MET MYC MYCL MYCN NTRK1 NTRK2 NTRK3 PDGFRA PDGFRB PIK3CB PIK3CA PPARG RICTOR TERT*

51 Gene fusions: *AKT2 ALK AR AXL BRCA1 BRCA2 BRAF CDKN2A EGFR ERBB2 ERBB4 ERG ESR1 ETV1 ETV4 ETV5 FGFR1 FGFR2 FGFR3 FGR FLT3 JAK2 KRAS MDM4 MET MYB MYBL1 NF1 NOTCH1 NOTCH4 NRG1 NTRK1 NTRK2 NTRK3 NUTM1 PDGFRA PDGFRB PIK3CA PRKACA PRKACB PTEN PPARG RAD51B RAF1 RB1 RELA RET ROS1 RSPO2 RSPO3 TERT*

(B) Oncomine Tumor Mutation Load Assay^®^ (TML), (ThermoFisher Scientific, Waltham, MA, USA):

The Oncomine Tumor Mutation Load Assay is a large, targeted NGS assay that has been designed to provide clinical researchers with an accurate assessment of TMB (mutations/Mb) in limited formalin-fixed, paraffin-embedded (FFPE) samples. This assay covers 1.65 Mb across 409 oncogenes that are relevant to the major cancer types.

409 genes: *ABL1 ABL2 ACVR2A ADAMTS20 AFF1 AFF3 AKAP9 AKT1 AKT2 AKT3 ALK APC AR ARID1A ARID2 ARNT ASXL1 ATF1 ATM ATR ATRX AURKA AURKB AURKC AXL BAI3 BAP1 BCL10 BCL11A BCL11B BCL2 BCL2L1 BCL2L2 BCL3 BCL6 BCL9 BCR BIRC2 BIRC3 BIRC5 BLM BLNK BMPR1A BRAF BRD3 BRIP1 BTK BUB1B CARD11 CASC5 CBL CCND1 CCND2 CCNE1 CD79A CD79B CDC73 CDH1 CDH11 CDH2 CDH20 CDH5 CDK12 CDK4 CDK6 CDK8 CDKN2A CDKN2B CDKN2C CEBPA CHEK1 CHEK2 CIC CKS1B CMPK1 COL1A1 CRBN CREB1 CREBBP CRKL CRTC1 CSF1R CSMD3 CTNNA1 CTNNB1 CYLD CYP2C19 CYP2D6 DAXX DCC DDB2 DDIT3 DDR2 DEK DICER1 DNMT3A DPYD DST EGFR EML4 EP300 EP400 EPHA3 EPHA7 EPHB1 EPHB4 EPHB6 ERBB2 ERBB3 ERBB4 ERCC1 ERCC2 ERCC3 ERCC4 ERCC5 ERG ESR1 ETS1 ETV1 ETV4 EXT1 EXT2 EZH2 FAM123B FANCA FANCC FANCD2 FANCF FANCG FAS FBXW7 FGFR1 FGFR2 FGFR3 FGFR4 FH FLCN FLI1 FLT1 FLT3 FLT4 FN1 FOXL2 FOXO1 FOXO3 FOXP1 FOXP4 FZR1 G6PD GATA1 GATA2 GATA3 GDNF GNA11 GNAQ GNAS GPR124 GRM8 GUCY1A2 HCAR1 HIF1A HLF HNF1A HOOK3 HRAS HSP90AA1 HSP90AB1 ICK IDH1 IDH2 IGF1R IGF2 IGF2R IKBKB IKBKE IKZF1 IL2 IL21R IL6ST IL7R ING4 IRF4 IRS2 ITGA10 ITGA9 ITGB2 ITGB3 JAK1 JAK2 JAK3 JUN KAT6A KAT6B KDM5C KDM6A KDR KEAP1 KIT KLF6 KRAS LAMP1 LCK LIFR LPHN3 LPP LRP1B LTF LTK MAF MAFB MAGEA1 MAGI1 MALT1 MAML2 MAP2K1 MAP2K2 MAP2K4 MAP3K7 MAPK1 MAPK8 MARK1 MARK4 MBD1 MCL1 MDM2 MDM4 MEN1 MET MITF MLH1 MLL MLL2 MLL3 MLLT10 MMP2 MN1 MPL MRE11A MSH2 MSH6 MTOR MTR MTRR MUC1 MUTYH MYB MYC MYCL1 MYCN MYD88 MYH11 MYH9 NBN NCOA1 NCOA2 NCOA4 NF1 NF2 NFE2L2 NFKB1 NFKB2 NIN NKX2-1 NLRP1 NOTCH1 NOTCH2 NOTCH4 NPM1 NRAS NSD1 NTRK1 NTRK3 NUMA1 NUP214 NUP98 PAK3 PALB2 PARP1 PAX3 PAX5 PAX7 PAX8 PBRM1 PBX1 PDE4DIP PDGFB PDGFRA PDGFRB PER1 PGAP3 PHOX2B PIK3C2B PIK3CA PIK3CB PIK3CD PIK3CG PIK3R1 PIK3R2 PIM1 PKHD1 PLAG1 PLCG1 PLEKHG5 PML PMS1 PMS2 POT1 POU5F1 PPARG PPP2R1A PRDM1 PRKAR1A PRKDC PSIP1 PTCH1 PTEN PTGS2 PTPN11 PTPRD PTPRT RAD50 RAF1 RALGDS RARA RB1 RECQL4 REL RET RHOH RNASEL RNF2 RNF213 ROS1 RPS6KA2 RRM1 RUNX1 RUNX1T1 SAMD9 SBDS SDHA SDHB SDHC SDHD SEPT9 SETD2 SF3B1 SGK1 SH2D1A SMAD2 SMAD4 SMARCA4 SMARCB1 SMO SMUG1 SOCS1 SOX11 SOX2 SRC SSX1 STK11 STK36 SUFU SYK SYNE1 TAF1 TAF1L TAL1 TBX22 TCF12 TCF3 TCF7L1 TCF7L2 TCL1A TET1 TET2 TFE3 TGFBR2 TGM7 THBS1 TIMP3 TLR4 TLX1 TNFAIP3 TNFRSF14 TNK2 TOP1 TP53 TPR TRIM24 TRIM33 TRIP11 TRRAP TSC1 TSC2 TSHR UBR5 UGT1A1 USP9X VHL WAS WHSC1 WRN WT1 XPA XPC XPO1 XRCC2 ZNF384 ZNF521.*

(C) Oncomine Solid Tumor DNA panel^®^, (ThermoFisher Scientific, Waltham, MA, USA):

Oncomine Solid Tumor Kits are next-generation sequencing (NGS) kits for both DNA and RNA workflows that enable the analysis of formalin-fixed and paraffin-embedded (FFPE) solid tumor samples. These kits provide accurate and reliable results for both DNA and RNA analyses, starting with as little a sample as 10 ng nucleic acid. With content coverage designed by leading clinicians from the OncoNetwork Consortium, these kits enable the assessment of actionable mutations that are involved in solid tumors, particularly those originating from the lungs and colon.

22 Hotspot genes: AKT1 ALK BRAF CTNNB1 DDR2 EGFR ERBB2 ERBB4 FBXW7 FGFR1 FGFR2 FGFR3 KRAS MAP2K1 MET NOTCH1 NRAS PIK3CA PTEN SMAD4 STK11 TP53

(D) KSBL Hotspot Panel^®^ (HOP), (Institute of Pathology of Liestal, Switzerland):

The KSBL Hotspot Panel is a custom panel designed by the Institute of Pathology of Liestal, based on the Ion Ampliseq Custom DNA panels (Thermo Fisher Scientific, Waltham, MA, USA). It partially covers 124 genes for hotspot alterations and 34 genes for copy number variations.

124 Hotspot genes: *AKT1 ALK APC AR ARAF ARID1A ATM ATR BAP1 BIRC2 BRAF BRCA1 BRCA2 BTK CBL CCND1 CCND2 CCND3 CCNE1 CDH1 CDK4 CDK6 CDKN2A CHEK2 CREBBP CSF1R CTNNB1 DCUN1D1 DDR2 EGFR ERBB2 ERBB3 ERBB4 ERCC2 ESR1 EZH2 FANCA FANCD2 FBXW7 FGF3 FGFR1 FGFR2 FGFR3 FGFR4 FLT3 FOXL2 GATA2 GNA11 GNAQ GNAS H3F3A HIST1H3B HNF1A HRAS IDH1 IDH2 IGF1R JAK1 JAK2 JAK3 KDR KIT KNSTRN KRAS MAP2K1 MAP2K2 MAP2K4 MAPK1 MAX MDM2 MDM4 MED12 MET MLH1 MLL2 MLL3 MSH2 MSH6 MTOR MYC MYCN MYD88 NF1 NF2 NFE2L2 NOTCH1 NOTCH2 NPM1 NRAS PALB2 PBRM1 PDGFRA PIK3CA PIK3CB PIK3R1 POLE PPP2R1A PTCH1 PTEN PTPN11 RAC1 RAF1 RB1 RET RHOA RNF43 ROS1 SETD2 SF3B1 SMAD4 SMARCA4 SMARCB1 SMO SPOP SRC STAT3 STK11 TERT TGFBR2 TP53 TSC1 U2AF1 VHL XPO1*.

34 Copy number variations: *ALK APC AR ATM BRAF CCND1 CDK4 CDK6 CDKN2A DDR2 EGFR ERBB2 ERBB4 FGFR1 FGFR2 FGFR3 FGFR4 IGF1R KDR KIT KRAS MDM2 MDM4 MET MTOR MYC MYCN PDGFRA PIK3CA PTEN RB1 SMAD4 TERT TP53*.

(E) Ion Torrent™ Oncomine™ cfDNA Assays (ThermoFisher Scientific, Waltham, MA, USA):

These are tumor type–specific, multi-biomarker next-generation sequencing (NGS) assays that enable the detection of somatic mutations down to a level of 0.1%, with >98% specificity for genes in plasma samples. The Oncomine cfDNA Assays and the Ion S5™ Systems enable tumor heterogeneity and reoccurrence research studies from minimal input DNA. The Oncomine cfDNA Assays include targets identified by the Oncomine™ Knowledgebase (a cancer genomics data resource) and reviewed by trained professionals.

○ Oncomine Lung cfDNA Assay^®^ covers 11 Hotspot genes

11 Hotspot genes: *ALK BRAF EGFR ERBB2 KRAS MAP2K1 MET NRAS PIK3CA ROS1 TP53*.

○ Oncomine Colon cfDNA Assay^®^ (cfColon) covers 14 hotspot genes

14 Hotspot genes: *AKT1 APC BRAF CTNNB1 EGFR ERBB2 FBXW7 GNAS KRAS MAP2K1 NRAS PIK3CA SMAD4 TP53*.
